# Effective and precise adenine base editing in mouse zygotes

**DOI:** 10.1007/s13238-018-0566-z

**Published:** 2018-07-31

**Authors:** Puping Liang, Hongwei Sun, Xiya Zhang, Xiaowei Xie, Jinran Zhang, Yaofu Bai, Xueling Ouyang, Shengyao Zhi, Yuanyan Xiong, Wenbin Ma, Dan Liu, Junjiu Huang, Zhou Songyang

**Affiliations:** 1 Key Laboratory of Gene Engineering of the Ministry of Education, Guangzhou Key Laboratory of Healthy Aging Research and SYSU-BCM Joint Research Center, School of Life Sciences, Sun Yat-sen University, Guangzhou, 510275 China; 2 Key Laboratory of Reproductive Medicine of Guangdong Province, School of Life Sciences and the First Affiliated Hospital, Sun Yat-sen University, Guangzhou, 510275 China; 3 State Key Laboratory of Ophthalmology, Zhongshan Ophthalmic Center, Sun Yat-sen University, Guangzhou, 510060 China; 4 Verna and Marrs Mclean Department of Biochemistry and Molecular Biology, Baylor College of Medicine, One Baylor Plaza, Houston, TX 77030 USA

**Dear Editor,**

Many human genetic diseases are caused by pathogenic single nucleotide mutations. Animal models are often used to study these diseases where the pathogenic point mutations are created and/or corrected through gene editing (e.g., the CRISPR/Cas9 system) (Komor et al., 2017; Liang et al., 2017). CRISPR/Cas9-mediated gene editing depends on DNA double-strand breaks (DSBs), which can be of low efficiency and lead to indels and off-target cleavage (Kim et al., 2016). We and others have shown that base editors (BEs) may represent an attractive alternative for disease mouse model generation (Liang et al., 2017; Kim et al., 2017). Compared to CRISPR/Cas9, cytidine base editors (CBEs) can generate C•G to T•A mutations in mouse zygotes without activating DSB repair pathways (Liang et al., 2017; Kim et al., 2017; Komor et al., 2016). In addition, CBEs showed much lower off-targets than CRISPR/Cas9 (Kim et al., 2017), making the editing process potentially safer and more controllable. Recently, adenine base editors (ABEs) that were developed from the tRNA-specific adenosine deaminase (TADA) of *Escherichia coli* were also reported (Gaudelli et al., 2017). As a RNA-guided programmable adenine deaminase, ABE can catalyze the conversion of A to I. Following DNA replication, base I is replaced by G, resulting in A•T to G•C conversion (Gaudelli et al., 2017; Hu et al., 2018). The development of ABEs has clearly expanded the editing capacity and application of BEs. Here, we tested whether ABEs could effectively generate disease mouse models, and found high efficiency by ABEs in producing edited mouse zygotes and mice with single-nucleotide substitutions.

Unlike CBEs that can generate premature stop codons with C-T conversion (TAG, TAA or TGA), ABEs cannot produce a new stop codon to disrupt gene function via A-G conversion. We therefore targeted mRNA splice sites in order to induce gene dysfunction. Since mammalian mRNA splicing requires a 5′ GU donor and a 3′ AG acceptor at intron-exon junctions, ABEs can block mRNA splicing and hence inactivate gene function by converting splice donors and acceptors to GC and GG. We named this strategy ABE-induced mRNA splicing defect (AI-MAST).

We first used ABE7.10 to target the mouse *Tyr* gene, whose dysfunction results in albinism in mice (Zhang et al., 2016). A gRNA was designed to target the splice donor at exon 3 of the *Tyr* gene, which is also predicted to be an ideal site for ABE. We then injected both ABE7.10 mRNA and the gRNA into mouse zygotes (Fig. S1A). Of the 20 embryos harvested 48 h later, 9 were edited (45.0%) with efficiencies ranging from 11.2% to 24.6% (Fig. S1B–D). In addition, 106 injected zygotes were transplanted into pseudopregnant mothers. Among the 23 pups obtained, 13 (56.5%) showed A-to-G editing with conversion frequencies of 14.6%–48.1% (Figs. S1B and S2), attesting to the feasibility of AI-MAST in generating point mutations in mice.

It should be noted that we did not obtain any white-coated F0 mice, likely due to insufficient A-to-G conversion rate at the splice donor site. However, when the T1–12 F0 mouse was mated with homozygous *Tyr* mutant (c.655G>T, *p*.*E219X*) C57BL/6J mice (Liang et al., 2017), 2/5 (40.0%) pups were albino (Fig. S3A). Sanger sequencing results indicated that the 2 albino pups were compound heterozygous for both the ABE target site and *Tyr* site (c.655G>T, *p*.*E219X*) (Fig. S3B), lending support to *Tyr* gene dysfunction as a result of A•T to G•C conversion at the splice donor of exon 3. Furthermore, analysis of RNAs extracted from the skin of these compound heterozygous mice found significant reduction of correctly spliced *Tyr* mRNAs compared with *Tyr*^E219X/+^ mice (Fig. S3C and S3D). Both *Tyr*^E219X/+^ and *Tyr*^E219X/E219X^ mice showed obvious reduction of *Tyr* mRNA, indicating that *Tyr*^E219X^ mutant RNA is subjected to degradation by nonsense-mediated mRNA decay (NMD). These data demonstrate that AI-MAST is capable of inducing mRNA splicing defects. However, whether phenotypes associated with mRNA splicing defects can be observed in F0 mice remains unknown.

To further explore one-step generation of disease mouse models using ABEs, we designed two gRNAs that targeted the splice sites at exons 61 and 66 of *Dmd* (Fig. 1A). These two sites were chosen because Dunchenne muscular dystrophy (DMD) remains a progressive neuromuscular degenerative disorder with no effective treatment. The largest in the human genome with 79 exons and 2.4 Mb long, the human *DMD* gene has recorded thousands of mutations (2,898 in the UMD-DMD database for DMD patients), including insertions, deletions, duplications and point mutations. At least 158 splice site mutations have been identified thus far, including the splice donor of exon 61 (c.9163+1G>A, GU-to-AU; c.9163+2T>G, GU-to-GG) and splice acceptor of exon 66 (c.9564-2A>T, AG-to-TG) (http://www.umd.be/DMD/4DACTION/W_DMDT1/9). Exons 61 and 66 are highly conserved between human and mouse, both at the genomic DNA (>91% similarity) and protein (100%) sequence level. With gRNA-1 and gRNA-2, 15/18 (83.3%) and 9/17 (52.9%) embryos were respectively edited (Fig. 1B). Allelic mutation frequencies in these embryos ranged from 10.1% to 88.7% (Fig. S4). Following transplantation of the gRNA-1 (270) and gRNA-2 (122) injected zygotes, 70.1% (47/67) pups were edited with varying allelic mutation frequency for gRNA-1, and 42.3% (11/26) pups were edited for gRNA-2 with a slightly lower allelic mutation frequency compared to gRNA-1 (Fig. 1B–E). While allelic mutation frequency differs among F0 mice, a total of 15 F0 mice were obtained that exhibited >95.0% frequency. Of the 67 F0 mice from group gRNA-1, 14 were edited with >95.0% efficiency (20.9%) (Fig. 1B). Remarkably, 5 of these 14 mice showed a rate of >99.0% (Fig. 1D). These 5 mice may in fact be pure mutant mice without any wild-type alleles, considering that error rates of deep sequencing may run up to 1% (Liang et al., 2017). In addition, one F0 mouse was edited with 98.0% efficiency from group gRNA-2 (Fig. 1E). These results combinedly suggest high editing efficiency of ABEs and the AI-MAST strategy in mouse zygotes.

**Figure 1:**
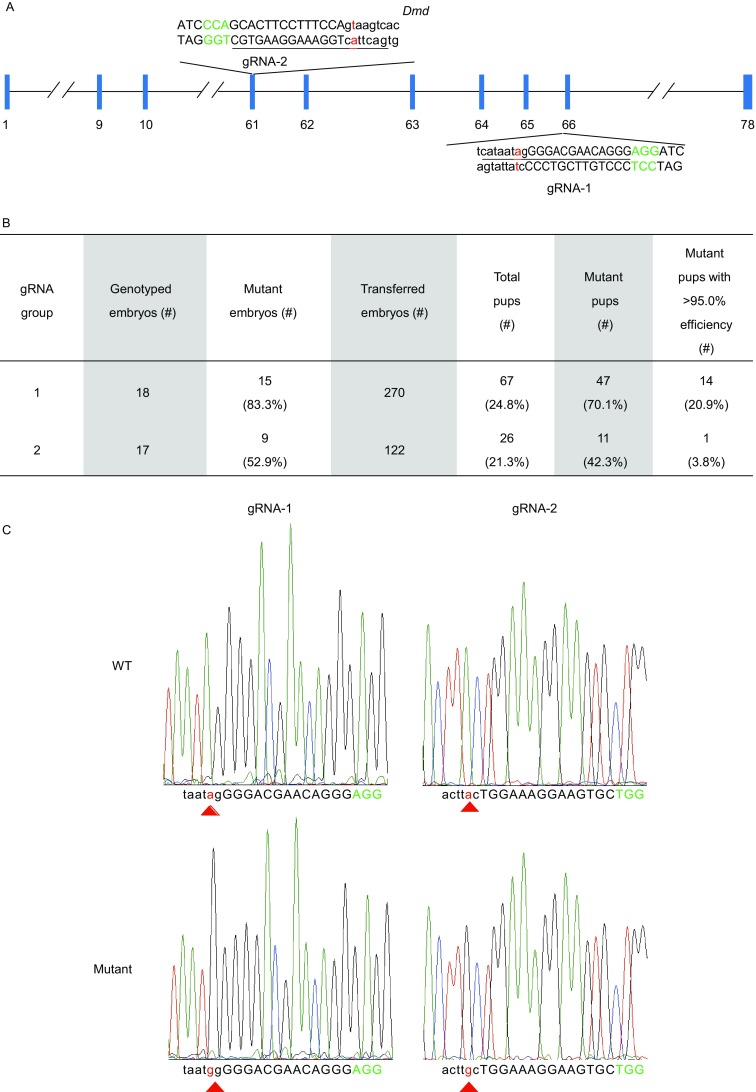

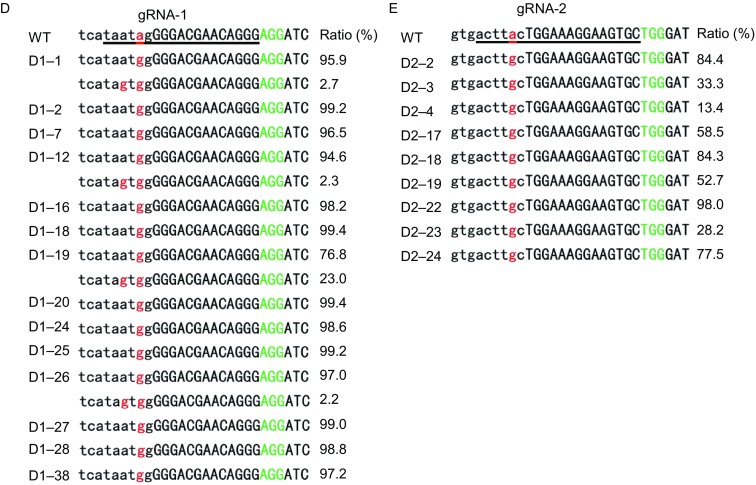
**Efficient targeting of the mouse** ***Dmd*** **gene by adenine base editors (ABEs)**. (A) Schematic representation of the two gRNA target sites in the *Dmd* gene locus. Exon-intron boundary sequences (both strands) are shown with exon sequences capitalized and intron sequences in lower case. The gRNA target sequence is underlined, with PAM in green and the adenine being mutated in red. (B) The number of injected and transplanted embryos and subsequent pup information for each gRNA group are listed in the table. (C) Representative Sanger sequencing chromatograms of PCR amplicons spanning each gRNA target site from wild-type (WT) vs. mutant mice (D1–12 and D2–22 for group gRNA-1 and gRNA-2 respectively). Red triangle marks the targeted/mutated adenine. (D) PCR amplicons spanning the gRNA-1 target site from the F0 newborns were analyzed by deep sequencing. Exons and introns are in capital and lower case letters respectively. Base substitutions, red. PAM, green. The frequency of each mutant allele within individual pups is listed on the right. (E) PCR amplicons spanning the gRNA-2 target site from the F0 newborns were analyzed by deep sequencing

We next sought to determine whether the ABE-induced *Dmd* splice site mutant mice would display DMD phenotypes. First, we quantified correctly spliced *Dmd* mRNAs by qPCR analysis of RNAs isolated from the quadriceps and hearts of mutant mice and their wild-type (WT) littermates (Fig. S5A). Correctly spliced *Dmd* mRNAs decreased significantly (>90%) in edited mice, indicating highly efficient base editing by AI-MAST (Figs. 2A and S5B). In addition, we were also able to detect incorrectly spliced transcripts that appeared to have used cryptic splice sites (Fig. S5C and S5D). Consistent with the drastic reduction in properly spliced mRNAs, the *Dmd* gene product (Dystrophin protein) was also nearly depleted in edited mice as assayed by immunostaining (Fig. 2B).

**Figure 2:**
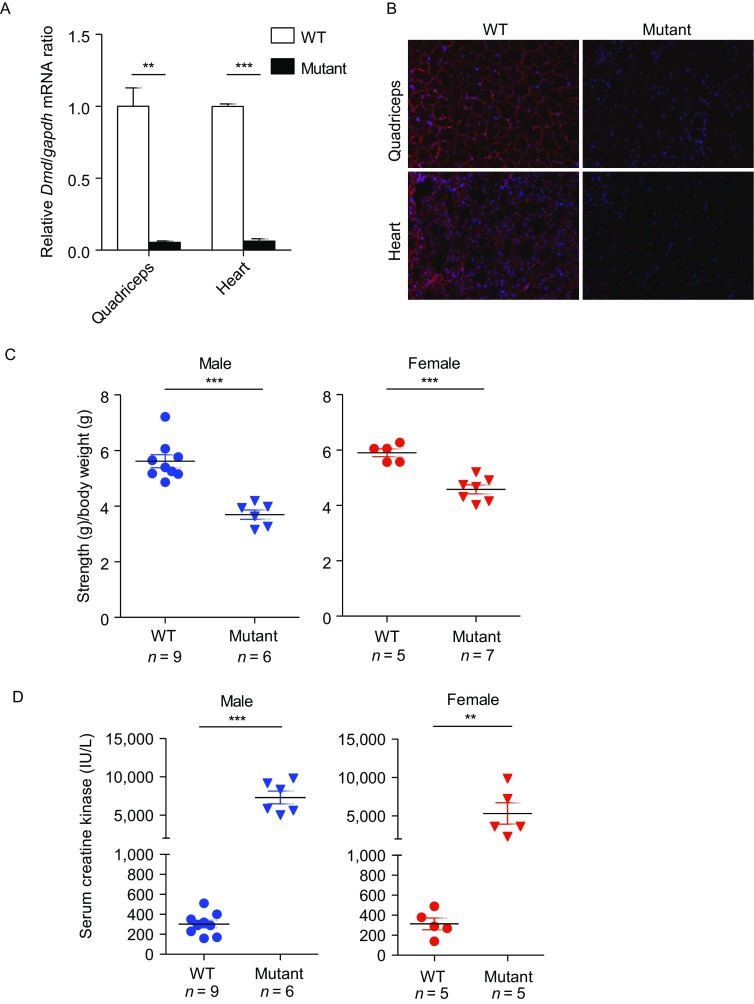

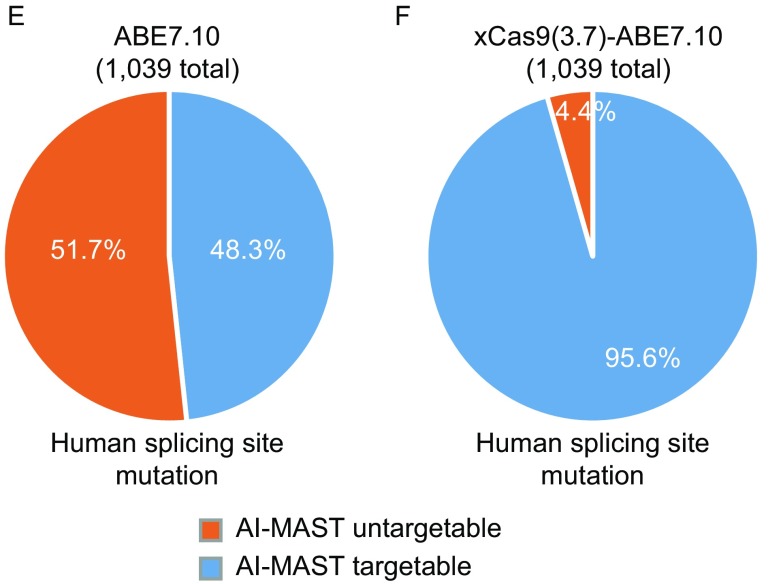
**One-step generation of DMD mice by ABE-induced mRNA splicing defect strategy (AI-MAST)**. (A) qPCR was carried out using RNAs extracted from the quadriceps and hearts of WT (D1–4, D1–14, D1–31) and mutant mice (D1–18, D1–25, D1–27) to quantify correctly spliced *Dmd* mRNAs. Data are presented as mean ± SEM (*n* = 3). **P < 0.01. ***P <0.001. Statistical significance was determined using the two-tailed Student’s *t*-test. (B) Immunofluorescence staining of Dmd in WT and mutant mice from (A). Representative images from pups D1–4 (WT) and D1–18 (mutant) were shown (*n* = 3). (C) Forelimb grip strength of 4–5 week old male and female mice from the *Dmd* gRNA-1 group was assessed using a force transducer. ***P < 0.001. Statistical significance was determined using the two-tailed *t*-test. (D) Serum creatine kinase levels in 4–5 week old male and female mice from the *Dmd* gRNA-1 group were determined. **P < 0.01, and ***P < 0.001. Statistical significance was determined using the two-tailed *t*-test. (E and F) Analysis of human disease splice site mutations that may be modeled by ABE7.10 (E) and xCas9(3.7)-ABE7.10 (F)

Dystrophin is necessary for muscle fiber strength, the absence of which results in muscle weakness. In humans, splicing defects in the *DMD* gene can cause Duchenne muscular dystrophy with severe symptoms including muscle fatigability and myocardial fibrosis (Birnkrant et al., 2018). Similarly, we also observed significant decreases in the forelimb grip strength of both male and female mutant mice (Figs. 2C, S6 and Table S1). Creatine kinase (CK) activity is a widely used marker in the investigation of skeletal muscle diseases (Birnkrant et al., 2018). In line with their muscle weakness, serum creatine kinase (CK) levels in these mutant mice were substantially elevated as well compared with WT controls, similar to muscular dystrophy phenotypes observed in *Dmd* mouse models (Fig. 2D and Table S2). When mated with WT mice, the A-to-G *Dmd* mutations in the mutant mice could be stably passed down to their progenies (Fig. S7). These data indicate that AI-MAST is suitable to establishing mouse models for human diseases in one step.

Off-target effects are a well-known problem of canonical CRISPR-Cas9 editing tools. To examine the rate of off-target deamination in F0 mice, we selected 10 mutant mice from each gRNA group for deep sequencing. For the top 5 predicted off-target sites of each gRNA (based on sequence similarity), no off-target deamination was found (Tables S3–5). Roughly 2/22 (9.1%) CBE-edited embryos and 3/57 (5.3%) CBE-edited F0 mice were found to contain alleles with indels (Liang et al., 2017; Kim et al., 2017). In comparison, we did not find any indels in ABE-edited embryos (33) or F0 mice (71). In addition, we found only A-to-G conversions, but no A-to-C/T conversions, which is in agreement with ABEs’ observed improved product purity in human cells (Gaudelli et al., 2017). Taken together, our data demonstrate that ABEs can efficiently and precisely convert base A to G in mouse embryos and represent a high-fidelity tool in generating point mutation mouse models.

We estimate that ~79.4% and 87.3% respectively of mouse and human protein-coding genes may be targeted by the AI-MAST strategy (Fig. S8A, S8B and Tables S6–7), suggesting broad applicability of AI-MAST in making gene deficiency mouse models and human cell lines. The recently developed xCas9 (3.7)-ABE7.10, an ABE variant with a broader PAM preference (5′-NGN-3′, 5′-GAA-3′, 5′-GAT-3′ and 5′-CAA-3′) (Hu et al., 2018) should further expand the target scope of our AI-MAST strategy (Fig. S8C, S8D and Tables S8–9). In humans, ~10% of pathogenic mutations in all Mendelian diseases comprise of splice site mutations (Faustino and Cooper, 2003), Our AI-MAST strategy therefore should prove particularly attractive in the generation of relevant animal models and the investigation of human diseases caused by splice-site defects. In-depth analysis revealed that 48.3% (517/1,039) of the human pathogenic mutations at splice sites can be generated by ABE7.10 (Fig. 2E and Table S10), and 95.6% can be generated by xCas9(3.7)-ABE7.10 (Fig. 2F and Table S11). In addition, we also found some conserved splice site mutations in human and mouse that can be generated by either ABE7.10 (64) or xCas9 (3.7)-ABE7.10 (163) (Tables S12 and S13). Working with human cell and mouse models of these mutation sites has the best chance of probing disease biology and developing possible new therapeutics.

While our manuscript was under review, two independent groups reported using ABEs to generate mouse models and repair disease mutations in adult mouse (Ryu et al., 2018; Liu et al., 2018). Our study together with the others not only highlight the fidelity and efficiency of ABEs in inducing A•T to G•C conversion, but also demonstrate their potential ease and versatility in generating disease models as well as correcting disease mutations in animal and human embryos (Liang et al., 2017).

## Electronic supplementary material

Below is the link to the electronic supplementary material.

Supplementary Script 1 (ZIP 17732 kb)

Supplementary Script 2 (ZIP 13862 kb)

Supplementary Material 3 (PDF 815 kb)

Supplementary Table 1 (XLSX 13 kb)

Supplementary Table 2 (XLSX 11 kb)

Supplementary Table 3 (XLSX 10 kb)

Supplementary Table 4 (XLSX 10 kb)

Supplementary Table 5 (XLSX 10 kb)

Supplementary Table 6 (XLSX 7994 kb)

Supplementary Table 7 (XLSX 8972 kb)

Supplementary Table 8 (XLSX 14218 kb)

Supplementary Table 9 (XLSX 16147 kb)

Supplementary Table 10 (XLSX 46 kb)

Supplementary Table 11 (XLSX 74 kb)

Supplementary Table 12 (XLSX 16 kb)

Supplementary Table 13 (XLSX 23 kb)

Supplementary Table 14 (XLSX 10 kb)

Supplementary Table 15 (XLSX 11 kb)
