# Supplementary material for: Effective and precise adenine base editing in mouse zygotes
Source: Protein Cell. 2018 Jul 31;9(9):808–13. doi: 10.1007/s13238-018-0566-z (PMC6107484; doi:10.1007/s13238-018-0566-z)
Supplement: Supplementary file 3 — Supplementary Material 3 (PDF 815 kb) [file 13238_2018_566_MOESM3_ESM.pdf]

Methods

Vectors. pcDNA3.1(-)-ABE7.10 was synthesized by Guangzhou IGE biotechnology LTD[11], and used for *in vitro* transcription. gRNAs were cloned into the pDR274 vector (Addgene). Oligos used for gRNA cloning are listed in Table S14.

Animals. All animal experiments were performed according to protocols approved by the Institutional Animal Care and Use Committee of School of Life Sciences, Sun Yat-sen University. Mice were housed under climate-controlled (22±1 °C) conditions in a designated pathogen-free animal facility with a 14-hour-light/10-hour-dark cycle. Superovulated C57BL/6J mice (6–8 week old females) were mated with C57BL/6 J males. Plugged females were sacrificed by cervical dislocation. Zygotes (0.5 day) were collected using potassium simplex optimized medium (KSOM) containing N'-2-Hydroxyethylpiperazine-N'-2-ethanesulfonic acid and sodium bicarbonate (HKSOM), and cultured in KSOM until genotyping or transplantation. Embryos were *in vitro* cultured for 48 h before genotyping or whole genome amplification. CD1 female mice (6–8 weeks old) that were mated with sterilized CD1 male mice were used as foster mothers.

***In vitro* transcription of gRNA and ABE7.10 mRNA.** gRNAs were transcribed using the MEGAshortscript T7 kit (Life Technologies) following the manufacturer's instruction. ABE7.10 mRNA was transcribed using the mMESSAGE mMACHINE T7 ULTRA kit (Life Technologies) following the manufacturer's instruction. The mixture of ABE3 mRNA (200 ng/μl) and gRNA (100 ng/μl) was then injected into the cytoplasm of 0.5-day mouse zygotes.

Genotyping of embryos and mice. Each embryo was transferred into a PCR tube containing 1 μl lysis buffer, and then incubated at 65°C for 3 h followed by 95°C for 10 min. The lysis product was then amplified by PCR. Mouse genotyping was done using a mouse genotyping kit (KAPA Biosystems, KK7351) following the manufacturer's instruction. Primers used are listed in Table S15.

Deep sequencing. PCR products were deep sequenced using the Illumina Hiseq 2000 as paired-end 250 reads. Primers used are listed in Table S13. The sequenced reads were aligned to reference sequences by BWA with default parameters (v0.7.13). Samtools (v1.3, http://samtools.sourceforge.net) and Picard tools (v2.2.2, http://picard.sourceforge.net) were used to build indices and sort reads. GATK (The Genome Analysis ToolKit, version 3.5) HaplotypeCaller and VarScan (v2.4.2, mpileup2snp and mpileup2indel with --min-reads2 10 --min-var-freq 0.01) were used to call variants for all samples and the combined variants of which were then divided into indels and SNVs by SelectVariants. The identified indels and SNVs were then used to generate

reference sequence to calculate the mutation rate. We then aligned the reference sequence to the reads of each barcode by Bowtie (version 1.1.2, http://bowtie-bio.sourceforge.net/index.shtml) with no mismatch.

qPCR. Total RNA from mouse quadriceps and hearts was extracted using Trizol (Invitrogen, 15596-018). First-strand cDNAs were synthesized using the RevertAid First Strand cDNA Synthesis Kit (Thermo Scientific, K1621) according to the manufacturer's instructions. qPCR was performed using the KAPA SYBR FAST Universal qPCR kit (KAPA Biosystems, KK4601) following the manufacturer's instruction. Primers used are listed in Table S15.

Serum creatine kinase (CK) test. Blood was collected from the submandibular vein of each mouse and then centrifuged at 3,000 g for 5 mins to collect the serum. Serum CK was measured using a chemistry analyzer (Hitachi, 7020).

Grip strength test. The body weight was assessed first to allow normalization for body weight. And then the tail was lifted so the mouse could grasp the grip strength meter (Yiyan Science technology LTD, YLS-13A). Measurements were repeated at least 5 times for each mouse.

Immunofluorescence staining. Mouse quadriceps and heart tissues were prepared by cryosection. Dystrophin was labeled using a rabbit anti-dystrophin antibody (Abcam, ab15277, 1:300), and detected using Alexa Fluor 555-conjugated donkey anti-rabbit IgG (ThermoFisher, A31572, 1:2,000). Samples were visualized using the Axio Observer D1 (Zeiss).

Western blot. Mouse quadriceps and hearts were homogenized in cold RIPA buffer (50 mM Tris-HCl pH8.0, 150 mM NaCl, 1% NP40, 1 mM EDTA pH8.0, 0.5% sodium deoxycholate, 0.1% SDS) supplemented with 0.2 mM phenylmethylsulfonyl fluoride and a proteinase inhibitor cocktail (Sigma, P8340), and centrifuged at 15,000 rpm for 15 min at 4°C. BCA Protein Assay Kit (Pierce, 23225) was used to determine protein concentration in the supernatant, and 60 µg protein lysates were used for western blot analysis with an anti-dystrophin antibody (Abcam, ab15277)(1:1,000), anti-GAPDH antibody (Santa Cruz, sc25778) (1:1,000), and the goat anti-rabbit secondary antibody (ThermoFisher, 65-6120) (1:5,000).

Identifying splice sites and splice site pathogenic mutations targetable by AI-MAST. The genome annotation files for human (GRCh37) and mouse (GRCm38) were downloaded from Ensembl (release75). First, all exon regions belonging to each gene were extracted and sorted by coordinates to obtain exon-intron/intron-exon junctions. Next, we used a computer program (Supplemental script 1) to design gRNA sequences around each junction. Take

the 5’-NGG-3’ PAM sequence as an example. If A appears 2bp upstream and GG 7-19bp downstream of a 5’ junction (intron-exon), or if T appears 2bp downstream and CC 5-17bp upstream of a 3’ junction (exon-intron), then a putative 30bp gRNA sequence that targets the junction site will be the output. The percentage of AI-MAST targetable genes was equal to the number of targetable gene divided by the total number of genes in the genome annotation file.

Data of pathogenic human mutations were downloaded from the ClinVar database. Pathogenic mutations at splice sites were collected using a computer program (Supplemental script 2), and those that could be targeted by Cas9-ABE7.10 or xCas9 (3.7)-ABE7.10 were identified using Supplemental script 1.

Figure S1

A

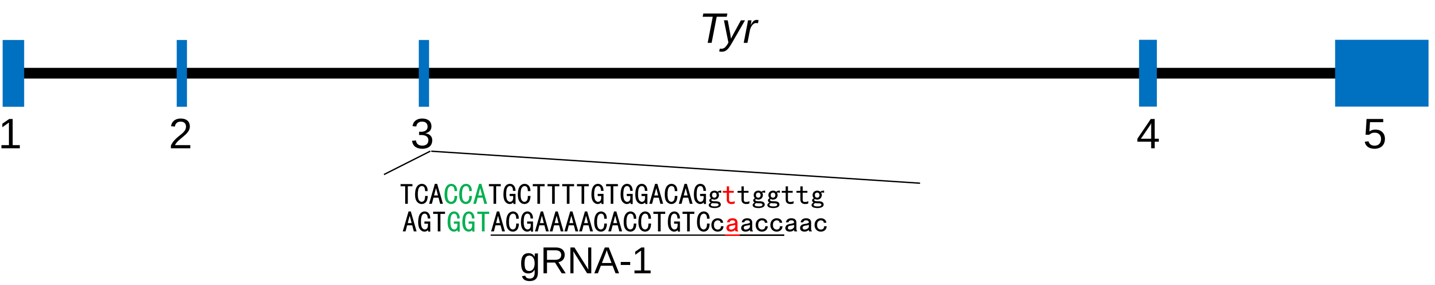

B

gRNA	Genotyped embryos (#)	Mutant embryos (#)	Transferred embryos (#)	Total pups (#)	Mutant pups (#)
1	20	9 (45.0%)	106	23 (21.7%)	13 (56.5%)

C

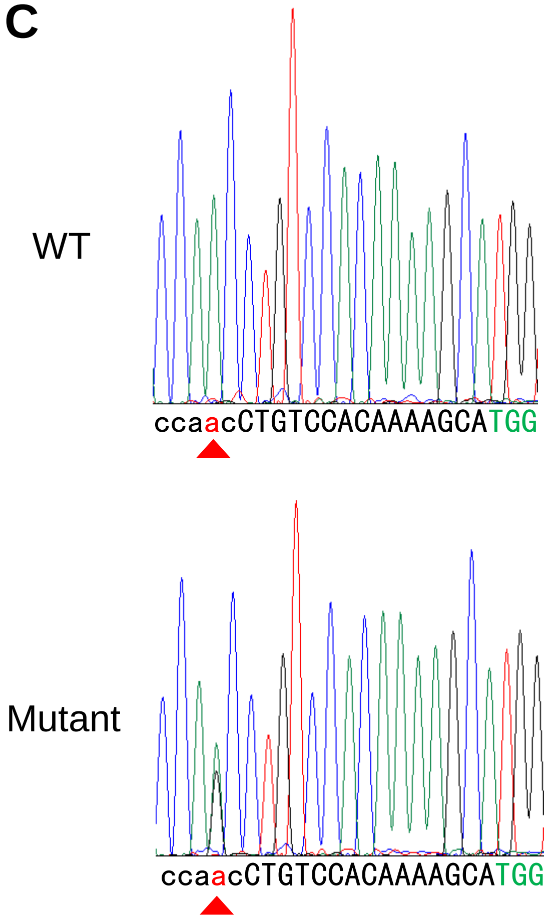

D

	gRNA-1	Ratio (%)
WT	caaccaacCTGTCCACAAAAGCATGGTGA	
#T1-7	caaccagcCTGTCCACAAAAGCATGGTGA	11.2
#T1-9	caaccagcCTGTCCACAAAAGCATGGTGA	19.7
#T1-11	caaccagcCTGTCCACAAAAGCATGGTGA	12.1
#T1-12	caaccagcCTGTCCACAAAAGCATGGTGA	24.6
#T1-13	caaccagcCTGTCCACAAAAGCATGGTGA	3.6
	caaccgacCTGTCCACAAAAGCATGGTGA	9.4
#T1-17	caaccagcCTGTCCACAAAAGCATGGTGA	2.8
	caaccgacCTGTCCACAAAAGCATGGTGA	16.8
#T1-18	caaccagcCTGTCCACAAAAGCATGGTGA	8.7
	caaccgacCTGTCCACAAAAGCATGGTGA	6.2
#T1-19	caaccagcCTGTCCACAAAAGCATGGTGA	20.5
#T1-20	caaccagcCTGTCCACAAAAGCATGGTGA	10.6
	caaccgacCTGTCCACAAAAGCATGGTGA	2.3

Supplementary Figure 1. Targeted A to G editing in the *Tyr* gene locus in mouse embryos by the adenine base editor 7.10 (ABE7.10).

(A) Schematic representation of the gRNA target site in the *Tyr* gene locus. Exon-intron boundary sequences (both strands) are shown with exon sequences capitalized and intron sequences in lower case. The gRNA target sequence is underlined, with PAM in green and the adenine being mutated in red.

(B) The number of injected and transplanted embryos and subsequent pup information are listed in the table.

(C) PCR amplicons spanning the *Tyr* target site from individual embryos were genotyped by Sanger sequencing following injection of the gRNA and ABE7.10 mRNA. Representative sequencing chromatographs for wild-type and mutant embryos are shown. Red triangles mark the targeted/mutated base.

(D) Sequences from various injected embryos were aligned. The gRNA target sequence is underlined. Intron sequences are in lower case. Exon sequences are capitalized. PAM sequence, green. Base substitutions, red. The frequency of each allele is shown on the right.

Figure S2

A

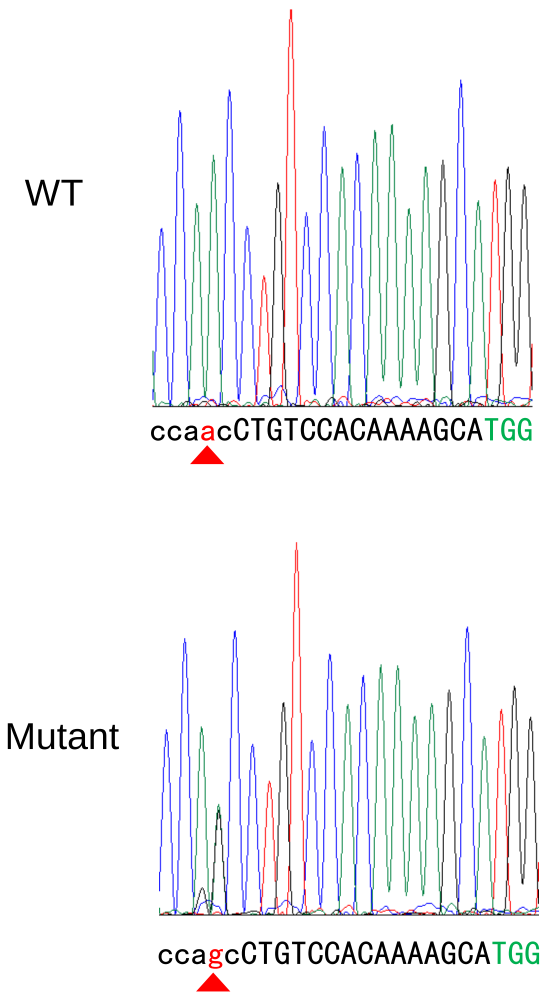

B

	gRNA-1	Ratio (%)
WT	caaccaacCTGTCCACAAAAGCATGGTGA	
T1-3	caaccgacCTGTCCACAAAAGCATGGTGA	4.5
	caaccggcCTGTCCACAAAAGCATGGTGA	10.5
T1-6	caaccagcCTGTCCACAAAAGCATGGTGA	19.8
	caaccgacCTGTCCACAAAAGCATGGTGA	5.1
	caaccggcCTGTCCACAAAAGCATGGTGA	4.2
T1-7	caaccggcCTGTCCACAAAAGCATGGTGA	21.3
T1-12	caaccagcCTGTCCACAAAAGCATGGTGA	39.1
	caaccgacCTGTCCACAAAAGCATGGTGA	9.0
T1-18	caaccgacCTGTCCACAAAAGCATGGTGA	35.2
T1-22	caaccagcCTGTCCACAAAAGCATGGTGA	1.3
	caaccgacCTGTCCACAAAAGCATGGTGA	1.7
	caaccggcCTGTCCACAAAAGCATGGTGA	11.6
T1-23	caaccgacCTGTCCACAAAAGCATGGTGA	39.4

Supplementary Figure 2. Targeted A to G editing in the *Tyr* gene locus in F0 mice by the adenine base editor 7.10 (ABE7.10).

(A) Representative Sanger sequencing chromatograms of PCR amplicons spanning the *Tyr* target site of wild-type (WT) and mutant mice (T1-12) are shown. Red triangle marks the targeted/mutated adenine.

(B) PCR amplicons spanning the *Tyr* target site from the F0 newborn mice were analyzed by deep sequencing. Exon and intron sequences are in capital letters and lower case respectively. Base substitutions, red. PAM, green. The frequency of each mutant allele within individual pups is listed on the right.

Figure S3

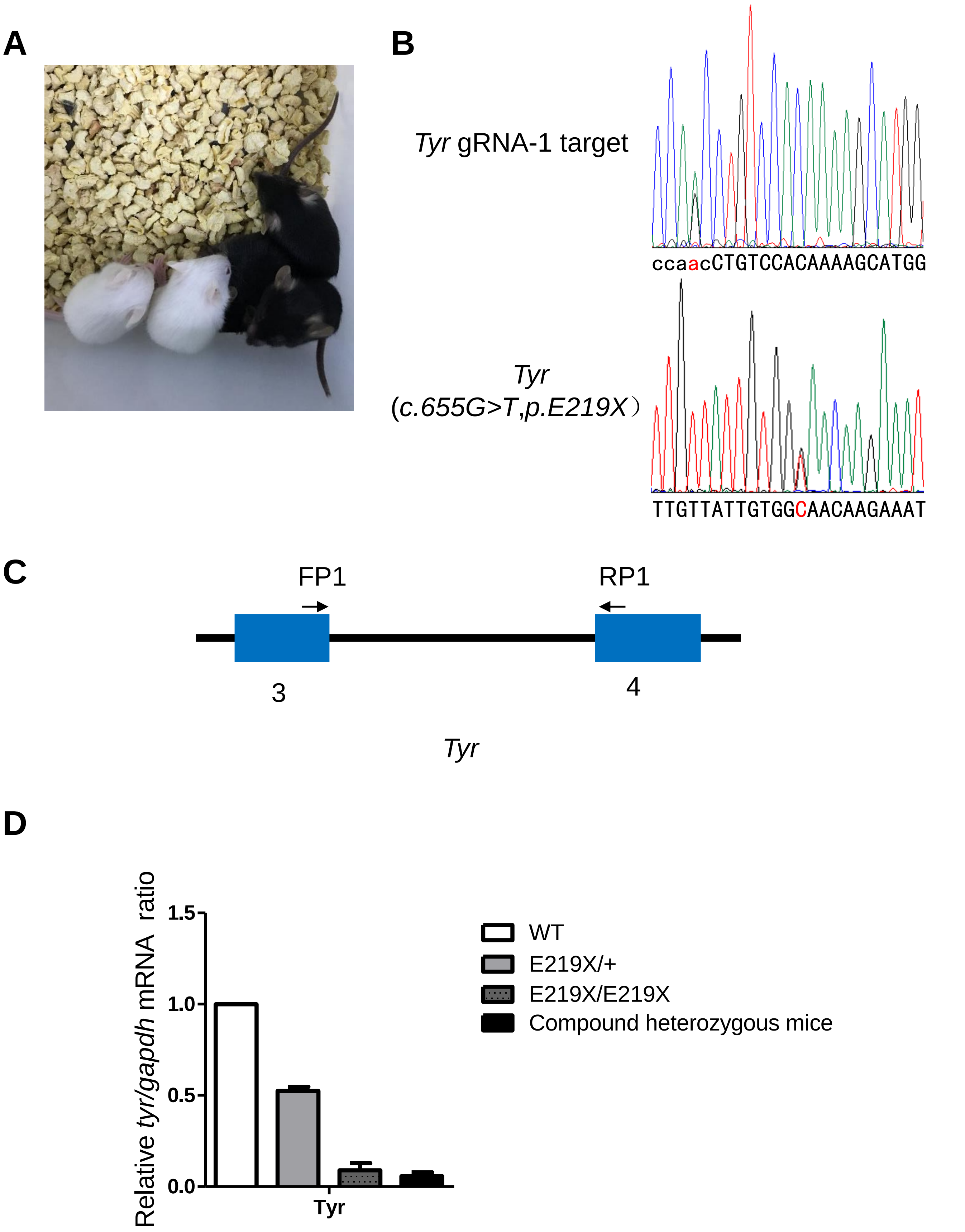
A
B
Tyr gRNA-1 target
ccaacCTGTCCACAAAAGCATGG
Tyr
(c.655G>T,p.E219X）
TTGTTATTGTGGCAACAAGAAAT
C
FP1
RP1
3
4
Tyr
D
Relative tyr/gapdh mRNA ratio
1.5
1.0
0.5
0.0
Tyr
WT
E219X/+
E219X/E219X
Compound heterozygous mice

Supplementary Figure 3. Genome editing using AI-MAST leads to mRNA splicing defects.

(A) Offspring of T1-12 F0 mouse and *Tyr* (*c.655G>T*, *p.E219X*) C57BL/6J albino mouse showed white coat color.

(B) Genotyping results of the albino pups at the *Tyr* gRNA-1 target site and *Tyr* (*c.655G>T*, *p.E219X*) site, indicating that the albino pups were compound heterozygous. Exon-intron boundary sequences are shown with exon sequences capitalized and intron sequences in lower case. The base being mutated is in red.

(C) Schematic diagram of the PCR strategy designed to quantify correctly spliced *Tyr* mRNAs in mice. FP1 & RP1 were used for qPCR, where the predicted size for PCR amplicons of correctly spliced mRNA is 116 bp.

(D) qPCR was carried out using RNAs extracted from the skin of WT, $Tyr^{E219X/+}$, $Tyr^{E219X/E219X}$ and compound heterozygous mice to quantify correctly spliced *Dmd* mRNAs. Data are presented as mean ± SEM (n=2).

Figure S4

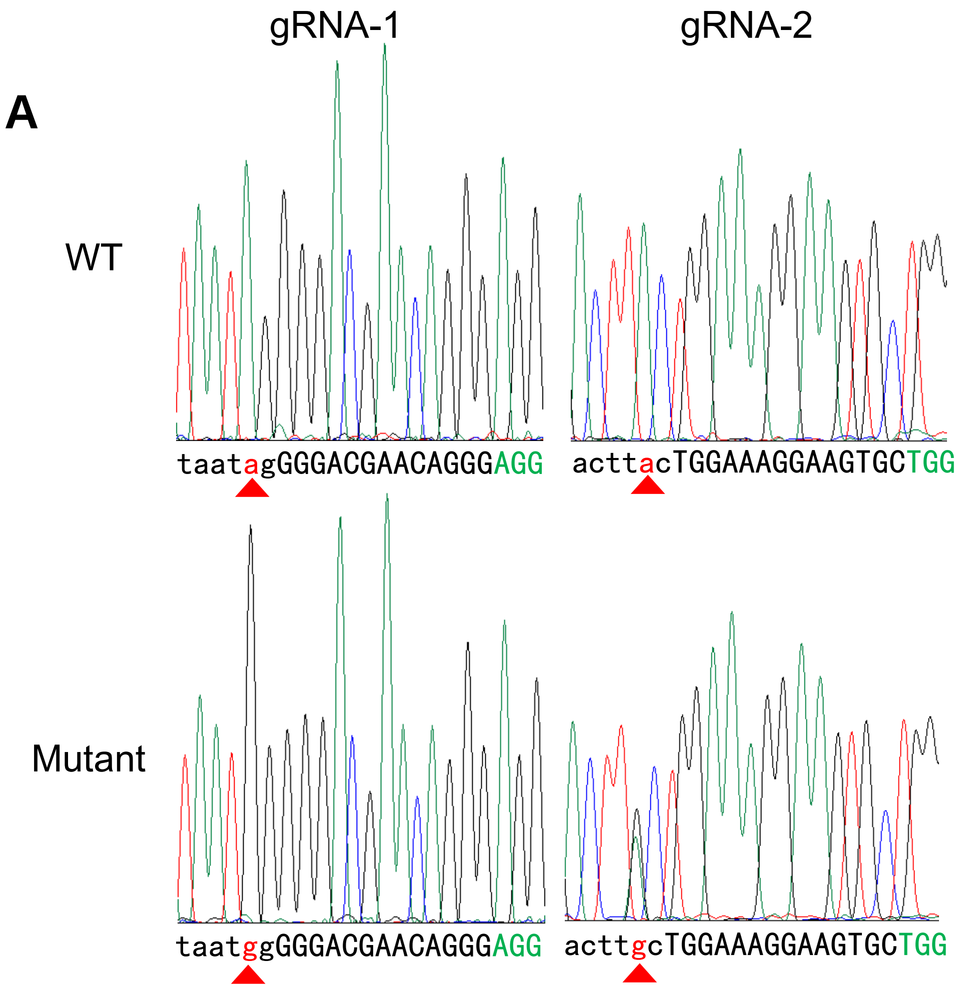

B

	gRNA-1	Ratio (%)
WT	tcataatagGGGACGAACAGGGAGGATC	
#D1-3	tcataatggGGGACGAACAGGGAGGATC	31.1
#D1-4	tcataatggGGGACGAACAGGGAGGATC	71.6
#D1-5	tcataatggGGGACGAACAGGGAGGATC	45.3
#D1-6	tcataatggGGGACGAACAGGGAGGATC	56.5
#D1-7	tcataatggGGGACGAACAGGGAGGATC	41.7
#D1-9	tcataatggGGGACGAACAGGGAGGATC	63.7
#D1-10	tcataatggGGGACGAACAGGGAGGATC	81.4
#D1-11	tcataatggGGGACGAACAGGGAGGATC	70.9
#D1-12	tcataatggGGGACGAACAGGGAGGATC	42.3
#D1-13	tcataatggGGGACGAACAGGGAGGATC	58.4
	tcatggtggGGGACGAACAGGGAGGATC	20.5
#D1-14	tcataatggGGGACGAACAGGGAGGATC	82.1
#D1-15	tcataatggGGGACGAACAGGGAGGATC	76.0
#D1-16	tcataatggGGGACGAACAGGGAGGATC	74.8
	tcatagtggGGGACGAACAGGGAGGATC	10.5
#D1-17	tcataatggGGGACGAACAGGGAGGATC	88.7
#D1-18	tcataatggGGGACGAACAGGGAGGATC	85.6

C

	gRNA-2	Ratio (%)
#WT	gtgacttacTGGAAAGGAAGTGCTGGGAT	
#D2-2	gtgacttgcTGGAAAGGAAGTGCTGGGAT	32.7
#D2-3	gtgacttgcTGGAAAGGAAGTGCTGGGAT	21.3
#D2-5	gtgacttgcTGGAAAGGAAGTGCTGGGAT	18.4
#D2-6	gtgacttgcTGGAAAGGAAGTGCTGGGAT	23.3
#D2-8	gtgacttgcTGGAAAGGAAGTGCTGGGAT	17.6
#D2-9	gtgacttgcTGGAAAGGAAGTGCTGGGAT	10.1
#D2-10	gtgacttgcTGGAAAGGAAGTGCTGGGAT	20.3
#D2-11	gtgacttgcTGGAAAGGAAGTGCTGGGAT	14.4
#D2-12	gtgacttgcTGGAAAGGAAGTGCTGGGAT	36.7

Supplementary Figure 4. Targeted A to G conversion in the *Dmd* gene locus in mouse embryos by ABE7.10.

(A) PCR amplicons spanning the *Dmd* target site from individual embryos were genotyped by Sanger sequencing following injection of the gRNA and ABE7.10 mRNA. Representative sequencing chromotagraphs for wild-type and mutant embryos are shown. Red triangles mark the targeted/mutated base.

(B and C) Sequences from various injected embryos for gRNA-1 (B) and gRNA-2 (C) were aligned.

The gRNA target sequence is underlined. Intron sequences are in lower case. Exon sequences are capitalized. PAM sequence, green. Base substitutions, red. The frequency of each allele is shown on the right.

Figure S5

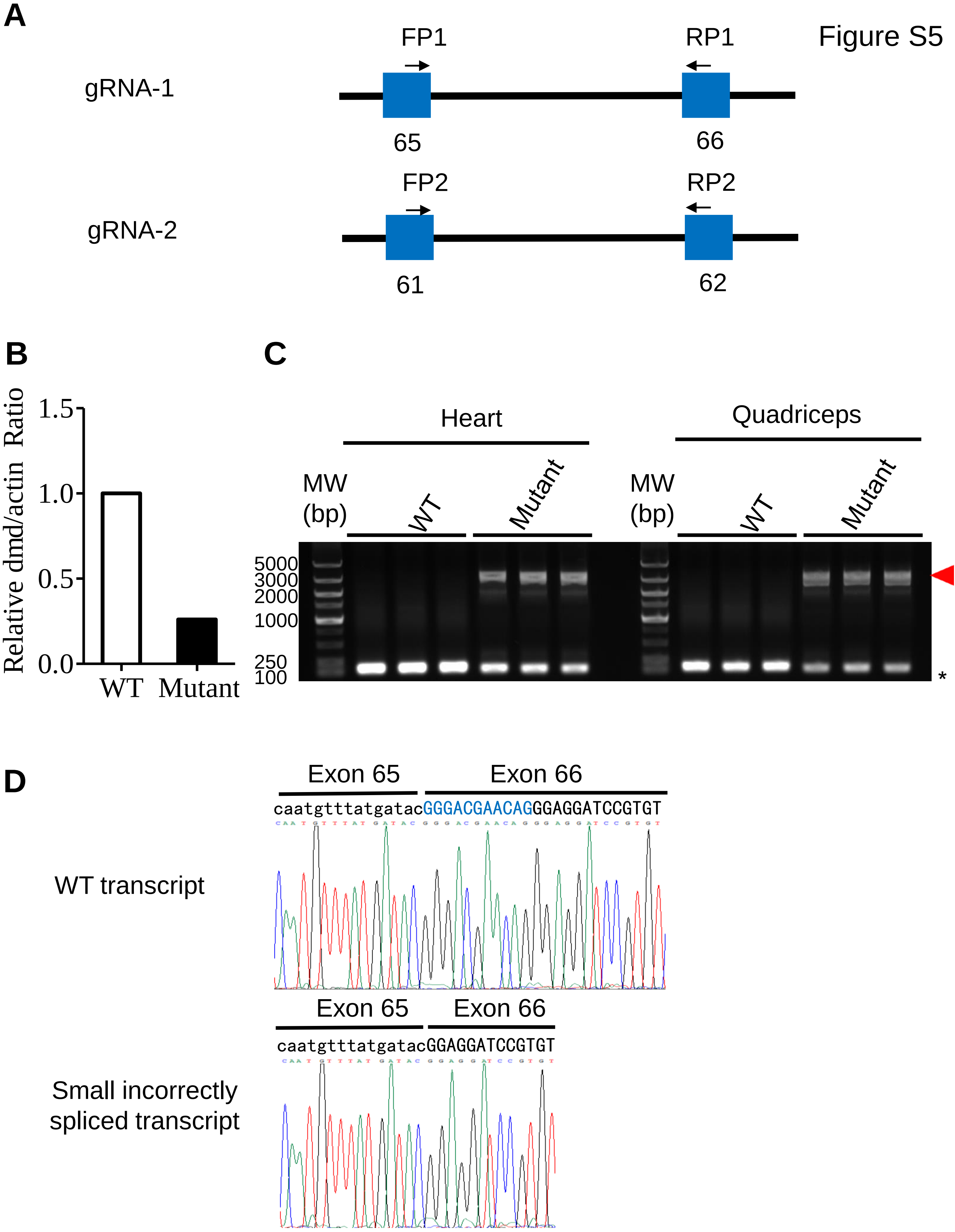
A
FP1
RP1
gRNA-1
65
66
FP2
RP2
gRNA-2
61
62
B
Relative dmd/actin Ratio
1.5
1.0
0.5
0.0
WT
Mutant
C
Heart
Quadriceps
MW (bp)
WT
Mutant
MW (bp)
WT
Mutant
5000
3000
2000
1000
250
100
*
D
WT transcript
Exon 65
Exon 66
caatgtttatgatacGGGACGAACAGGGAGGATCCGTGT
Small incorrectly spliced transcript
Exon 65
Exon 66
caatgtttatgatacGGAGGATCCGTGT

Supplementary Figure 5. Examining mRNA splicing in edited mice.

(A) Schematic diagram of the PCR strategy designed to quantify correctly spliced *Dmd* mRNAs in mice edited using the two gRNAs. FP, forward primer. RP, reverse primer. Numbers under blue boxes refer to the specific exons. For FP1 and RP1, the PCR amplicon of correctly spliced mRNA is 121 bp. For FP2 and RP2, the PCR amplicon of correctly spliced mRNA is 133 bp.

(B) Impaired splicing in *Dmd* gRNA-2 edited mutant mice. qPCR by FP2 and PR2 was used to quantify correctly spliced *Dmd* mRNA in 1 WT mouse and 1 mutant mouse edited by gRNA-2.

(C) RNA was isolated from the quadriceps and hearts of 3 WT mice and 3 mutant *Dmd* mice (D1-18, D1-25, and D1-27) from group gRNA-1 and used for RT-PCR analysis by FP1 and RP1. Clearly incorrectly spliced mRNA transcripts are indicated by red triangle. *: the lower band in the mutant mice lane was also from incorrectly spliced mRNA transcripts that appeared to have used cryptic splice acceptors.

(D) Sanger sequencing of the correctly spliced mRNA transcripts in WT mouse AND smaller incorrectly spliced mRNA transcripts in mutant mouse from (c). Sequences of exon 65 are in lower case. Sequences of exon 66 are capitalized. The deleted 11 nt sequence in the smaller incorrectly spliced transcript is in blue.

Figure S6

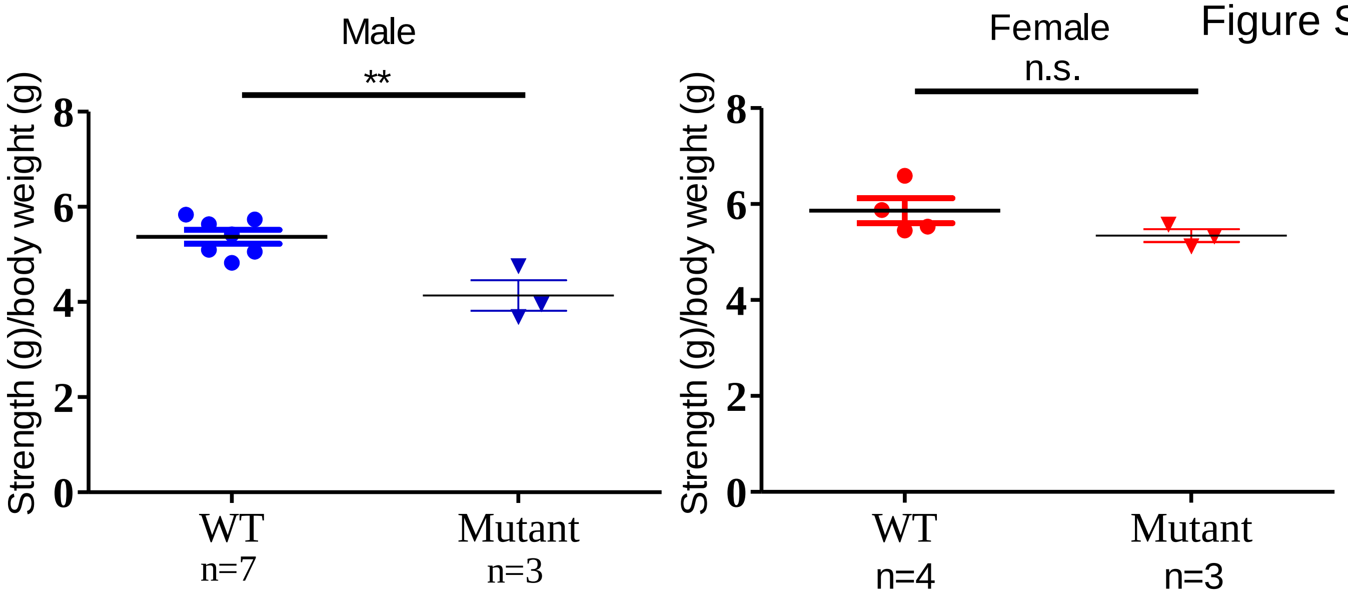

Supplementary Figure 6. Grip strength of *Dmd* gRNA-2 edited mutant mice. Body weight and forelimb grip strength of 8-week old WT and mutant mice were measured.

Figure S7

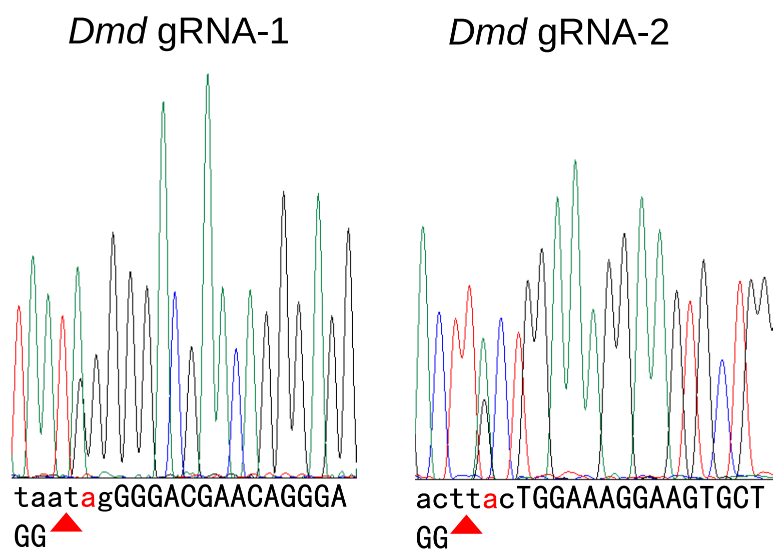

Supplementary Figure 7. Germline transmission of the *Dmd* mutation. Representative sequencing results of offspring from mutant F0 mice are shown here (D1-13 and D2-2 respectively)

Figure S8

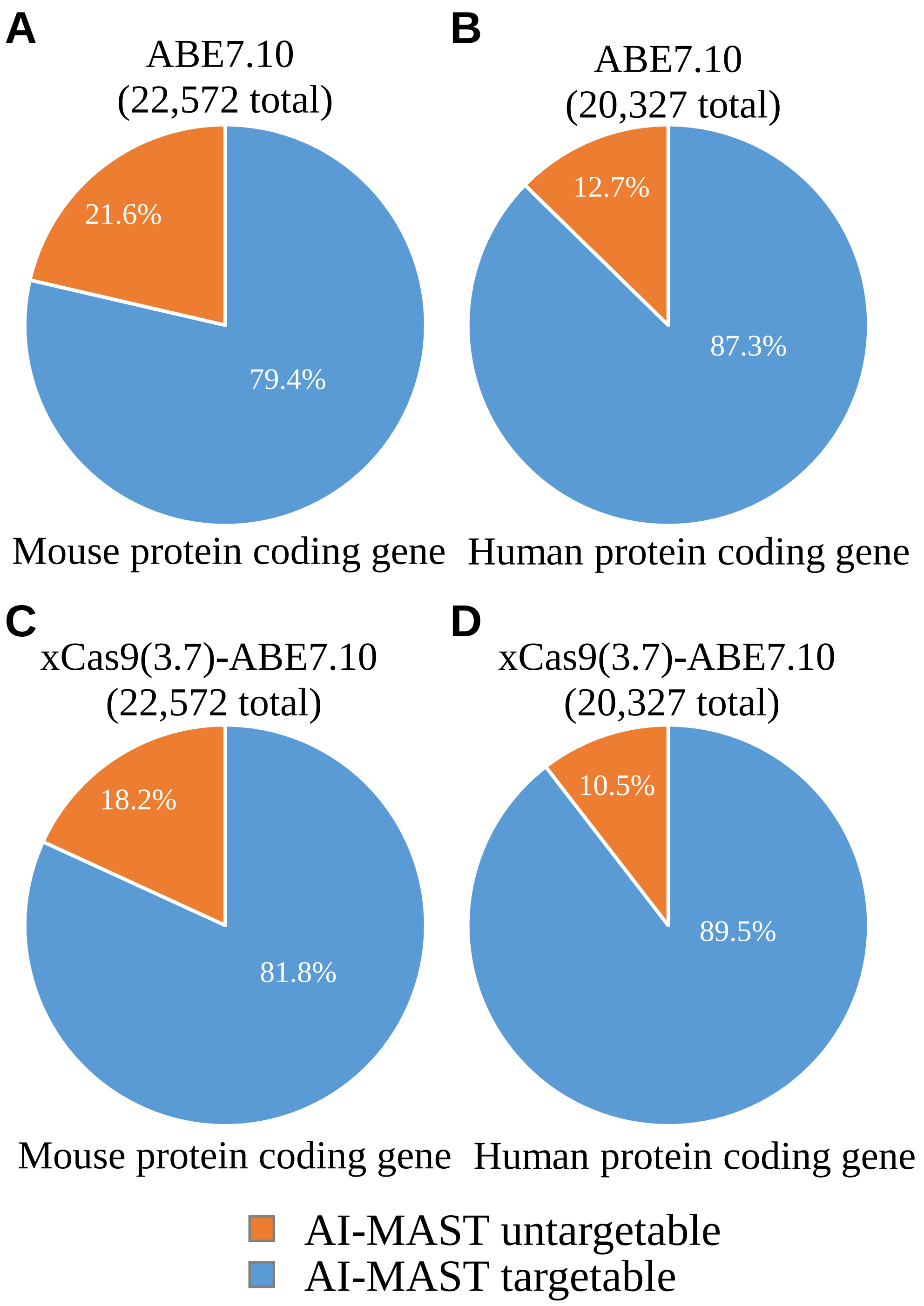

Supplementary Figure 8. Analysis of genes targetable by AI-MAST. ABE7.10 targetable mouse (A) and human (B) protein coding genes, and xCas9(3.7)-ABE7.10 targetable mouse (C) and human (D) protein coding genes are analyzed.
